# Life-Events Mediate the Prediction of Parental Alienation on Depression in Rural Left-Behind Children: A Longitudinal Study

**DOI:** 10.3389/fpsyt.2022.864751

**Published:** 2022-06-17

**Authors:** Xuemei Qin, Xiaoxiao Sun, Mengjia Zhang, Beijing Chen, Fei Xie, Zhaohua Chen, Sitong Shen, Chong Wen, Xiaomei Ren, Qin Dai

**Affiliations:** ^1^Department of Nursing, Army Medical University, Chongqing, China; ^2^Department of Psychology, Army Medical University, Chongqing, China

**Keywords:** alienation toward parents, depression, Hierarchical Linear Model, left-behind children, life-events

## Abstract

**Objectives:**

Long-time separation with parents during early life, such as left-behind children (LBC, one or both of whose parents are leaving for work for at least a period of 6 months), may contribute to high alienation toward parents and endanger their mental health (e.g., depression). However, the dynamic status of depression and potential prediction of alienation on depression in LBC remained largely unknown. This study aimed to examine the dynamic status of depression, prediction of alienation toward parents on later depression in rural LBC, and a potential mediation of life-events.

**Methods:**

A total of 877 LBC in rural areas of China were recruited and surveyed at five time-points (baseline, T0: 1-month, T1: 3-months, T2: 6-months, T3: 12-months, T4) with the Inventory of Alienation Toward Parents, Childhood Depression Inventory, and Adolescent Self-Rating Life-Events Checklist. The Hierarchical Linear Model (HLM) and Hayes's PROCESS macro model were conducted to estimate the developmental trend and hierarchical predictors of depression.

**Results:**

The left-behind children aged 9-years old experienced higher depression than the children with other ages. At baseline, the children in the family atmosphere of frequent quarrels and compulsive parenting style reported a higher level of alienation toward parents, life-events, and depression. Alienation toward parents, life-events, and depression were positively and moderately correlated with each other (*r* = 0.14 ~ 0.64). The HLM model depicted a linear decline in depression, alienation, and life-events with an average rate of 0.23, 0.24, and 0.86, respectively, during the five time-points. Also, T0 alienation toward parents and T0 life-events positively predicted the developmental trajectory of depression over time, and T0 life-events positively predicted the descendant rate of depression. Notably, life-events mediated the prediction of baseline alienation toward parents on T4 depression in LBC.

**Conclusion:**

This study is among the first to reveal that alienation toward parents predicts the developmental trajectory of later depression in LBC. The findings that life-events mediate the prediction of alienation on later depression further suggest the importance of family and social factors in the occurrence of depression in LBC. The findings warrant the necessity to consider the family and social factors when evaluating and reducing risks for mental health problems in LBC, i.e., relationship with parents (especially alienation toward parents) and life-events need further attention.

## Introduction

Along with the acceleration of industrialization and urbanization in China, a large number of rural labors migrate to prosperous cities with the hope of seeking better financial conditions for their families. Due to the household registration policy and other financial barriers, it is of great difficulty for migrant workers to solve the problems of education and medical care for their children in the place where they are employed; therefore, a large population of children are left behind in rural areas (1), named left-behind children (LBC). The LBC refers to the children under the age of 18, who are left at home for a period of at least 6 months with one or both of their parents leaving home for work (2). According to the 2015 survey conducted by the Chinese Ministry of Civil Affairs, the Ministry of Education and the Ministry of Public Security, the number of rural LBC in China are 40.51 million (3). The biggest problem of LBC faced is that their living condition lacks the companionship and care of parents, which may have a profound impact on their health and growth. A recent systematic review shows that 91 of 111 studies have reported poor health outcomes of LBC in China (4). In general, LBC are susceptible to many health problems (5), including emotional, behavioral, and mental health problems, such as depression (6, 7). The poor developmental conditions of LBC have attracted widespread attention in Chinese society currently.

Depression, a common negative emotion, may be expressed when people fail to cope with external pressure (7). A national survey of 10,123 adolescents aged 13–18 years in the USA found that lifetime and 12-month prevalence of major depressive disorder rates were 11.0% and 7.5%, respectively (8). In adolescence, the rates of depression increase substantially, and the estimated cumulative incidence in this population approximates the lifetime prevalence rate in adult (9). For LBC, the prevalence rates of depression in China have been reported with a range of 12.1–51.4% using different depression scales (10). Compared with the non-LBC, the LBC seem to be especially vulnerable to depression (10, 11). Moreover, it was found that the more serious symptom of depression, the higher occurrence of the non-suicidal self-injury in LBC (12). These findings suggest a great practical significance to observe the status of depression in LBC. In addition, the depression status in LBC might fluctuate due to a variety of influential factors (13). Thus, a multi-time-point investigation was warranted to observe the dynamic status of depression in LBC.

Higher levels of depression in LBC would be explained mainly by difficulties in establishing a good parent–child relationship because of the parent–child separation and the absence of parental care (7). In fact, a prolonged separation with parents during early life may increase the alienation toward parents in adolescents (14), in which alienation toward parents is defined as negative feelings, such as being emotional distant, possessive, even been controlled during the interaction with parents (15, 16). Compared with the non-LBC, the LBC tasted more and stronger feeling of alienation toward their parents (17, 18). Alienation toward parents may increase individual vulnerability to stress, and result in significant short- and long-term negative outcomes, such as depression and alienating from their own children (19). Consistently, the adolescents with a strong sense of alienation toward their parents were more susceptible to depressive symptoms (20). The retrospective studies further revealed that alienation toward parents caused various behavioral and emotional problems, such as depression in adults (21, 22). Importantly, the prior investigation confirmed a direct connection between alienation toward parents and depression in LBC (6). However, the prediction of alienation toward parents on depression in LBC still remains unexplored, which necessitates a longitudinal follow-up investigation.

The effect of alienation on depression may be mediated by other factors. Existing evidence show that the negative life-events positively relate to the incidence of depression at different ages (23, 24), which might be a possible risk mediator between alienation and later depression. Life-events refer to events or situations that threaten, challenge, exceed, or damage individual psychological or physical health (25). According to Erikson's psychosocial theory, parent–child relationship plays an important role in the process of children's growth; life-events may affect parents' behavior and parent–child relationship (26), which may boost the occurrence of adolescent depression. Adolescents with high levels of alienation toward parents may have problems in dealing with negative life-events effectively, while high levels of parent–child attachment can be buffering against the negative consequences (27). Moreover, the previous investigations indicated that LBC who were experiencing negative life-events had stronger alienation toward parents (6). They also showed stronger stress when experiencing negative events and exhibited more depressive symptoms than the non-LBC (28). Indeed, those painful experiences exposed to early parental loss or separation may increase youths' distance toward parents and heighten their reactivity to subsequent stressful life-events, and further result in depression (29). All these results indicated a connection between alienation toward parents and depression in adolescents and potential mediation of life-events. However, these effects were not tested directly, and none of these studies used longitudinal data. Thus, the mediation of life-events between predictive pathway from alienation to depression in LBC remains unsettled, which is potentially important to better understand and prevent the occurrence of depression in LBC.

To sum up, this study aims to reveal the dynamic status of depression in LBC with a longitudinal study, and further explores the prediction of alienation toward parents on later depression of LBC, and potential mediation of life-events. Specifically, the following hypotheses were proposed:

1) There may be a certain level of depression in LBC with a variation across the time.
2) There may be a positive prediction of alienation toward parents on the depression of LBC.
3) Life-events may mediate the prediction of alienation toward parents on the depression of LBC.

## Methods

### Participants

Students in fourth to sixth grade of primary school were eligible for this longitudinal investigation from rural area in Chongqing, China, in which the left-behind phenomenon is quite common. According to a survey in 2015, the proportion of LBC in rural Chongqing was estimated to be 76.37% (30). The inclusion criteria for sampling population included the following: 1) Children who can read and write Chinese. 2) One or both parents are leaving for work for a period that is longer than 6 months. Children with obvious physical or developmental disabilities were excluded. Children filled in self-rating scales about the alienation toward parents, depression, and life-events at five time-points (baseline, T0: 1-month, T1: 3-months, T2: 6-months, T3; and 12-months, T4). The reason that we chose students from fourth to sixth grade of primary school stemmed from the fact that the children in third grade or below had difficulties in understanding or answering some questions accurately (31). Among the 1,090 questionnaires collected with convenience sampling, 153 participants were the non-LBC. Sixty participants, who did not entirely complete all the measures or whose left-behind types changed during the four follow-up surveys, were excluded from the final analysis. The final sample size was reduced to 877. By applying the Wilcoxon rank–sum test (32), there was no significant difference in the scores of alienation toward parents (*Z* = –0.99, *p* = 0.324), life-events (*Z* = −0.40, *p* = 0.687), and depression (*Z* = −0.05, *p* = 0.960) between the exclusion subjects (*n* = 60) and the effective subjects (*n* = 877) who fully participated in the longitudinal survey. The median and interquartile range (IQR) age was 10 (10, 11) years. Of them, 466 were male, resulting in almost equal proportion of male and female.

### Instruments

Socio–demographic information, including gender, grade, age, family atmosphere (harmony, occasional quarrel, and frequent quarrel), parenting style (compulsive, indulged, spoiled, and democratic), and parents' marital status (un-divorced, divorced, father remarried, mother remarried, and both remarried), were collected.

Alienation toward parents was measured by the Inventory of Alienation toward Parents (IAP), which was designed in our previous work (18). This scale consists of 18 items assessing 2 dimensions covering maternal alienation (9 items; sample item: “I feel abandoned by my mother”) and paternal alienation (9 items; sample item: “I feel unable to communicate with my father”). All items are rated on a 5-point Likert scale from 1 (totally inconsistent) to 5 (totally consistent), and scores are calculated by summing all of the responses, with higher scores that mean higher level of alienation toward parents. This scale has shown good reliability and validity in the previous research (17). The Cronbach's alpha coefficients measured in this study were 0.871, 0.890, 0.889, 0.905; and 0.922 for the five surveys, respectively.

Depression was measured by the Childhood Depression Inventory (CDI) which was revised by Wu (33). It is used to assess depression symptoms in children aged 7 ~ 17 years, including 27 items and 5 dimensions covering anhedonia, negative emotions, low self-esteem, inefficient sense, and interpersonal problems. All items are rated on a 3-point Likert scale ranging from 0 (occasionally) to 2 (always). Scores are calculated by summing all of the responses, with higher scores indicating a higher level of depression. This scale has demonstrated a good reliability and validity in Chinese children (34). The Cronbach's alpha coefficients of this scale in this study were 0.869, 0.880, 0.887, 0.889; and 0.895 for the five surveys, respectively.

Stressful life-events were measured by the Adolescent Self-Rating Life-Events Checklist (ASLEC) (35). This measure consists of 27 items and 6 dimensions covering interpersonal relationship (5 items; e.g., “Been misunderstood or blamed”), study pressure (5 items; e.g., “Heavy learning burden”), punishment (7 items; e.g., “Been criticized or punished”), losing (3 items; e.g., “Bereavement”), healthy adaptation (4 items; e.g., “Prolonged separation with families”), and something else (4 items; e.g., “Fighting with people”). Items are rated on a 6-point Likert scale ranging from 0 (none) to 5 (extremely serious), and higher total scores reflect more life-events. The ASLEC has shown good reliability and validity in the previous research (36). The Cronbach's alpha coefficients in this study were 0.916, 0.924, 0.927, 0.933, and 0.936 for the five surveys, respectively.

### Procedure

Written informed consent was obtained from both students and their parents, which was approved by the Human Research Ethics Committee, Army Medical University. The researchers explained the study to children and their parents in written and verbal. After this, children applied for participation independently. The self-report questionnaires were investigated in the classrooms of primary school at five time-points (baseline, T0: 1-month, T1: 3-months, T2: 6-months, T3: 12-months, T4): IAP, CDI, and ASLEC. The children were debriefed and received incentives after the fifth survey.

### Analysis

The scores of depression, life-events, and alienation were described as median and interquartile range (IQR) since the variables were not normally distributed, and the categorical variables were described by number and percentages. The independent-sample Mann–Whitney U test as well as the Kruskal–Wallis test were used to compare the differences on the scores of alienation toward parents, life-events, and depression among the different demographic variables. Spearman correlation was carried out to examine the association between alienation toward parents, life-events, and depression.

The Hierarchical Linear Model (HLM) was further utilized to analyze the developmental trend of depression, and the prediction of alienation and life-events at baseline on later depression. The HLM is basically consisted of a null model, an unconditional model, and a full model. 1) The null model, a model including depression at five different test time-points as the outcome variable without any predictive variables, was mainly applied to judge whether there was a hierarchical structure in the developmental trajectory of depression, i.e., whether the data was suitable for HLM analysis. 2) An unconditional model was constructed to estimate whether the developmental trajectory of depression in LBC was linear. According to the analysis principle of the HLM (37), time was considered as an independent variable of Level 1 (first to fifth investigation time-point), and the developmental trajectory of depression during five time-points were considered as the outcome variable. Then, an unconditional linear growth model was built. On the one hand, the model can judge whether time (variable of Level 1) has a significant effect on the developmental trajectory of depression; on the other hand, it can further determine whether variables of Level 1 are significantly affected by Level 2, and if so, appropriate variables should be introduced in Level 2 for further analysis in the full model. The Level 1 model was represented by the equation, Depression = B_0_ + B_1_ × (Time) + R. 3) The full model utilized the Level 1 model to observe the influence of the time (within-individual variable) on the developmental trajectory of depression, and the Level 2 model was used to investigate the effect of the between-individual variables (alienation toward parents, life-events) or the combined effect (alienation toward parents and life-events) on the developmental trajectory of depression. Level 2 variables centered on their grand means and the following two variables were included: T0 alienation toward parents and T0 life-events, with age, family atmosphere, and parenting style as controlled variables. The full model was represented as the following equation: Level 1 Model: Depression = B_0_ + B_1_ × (Time) + R; Level 2 Model: B_0_ = γ_00_ + γ_01_ × (T0 Age) + γ_02_ × (T0 Family atmosphere) + γ_03_ × (T0 Parenting style) + γ_04_ × (T0 Alienation toward parents) +γ_05_ × (T0 Life-events) + U_0_; B_1_ = B_10_ + B_11_ × (T0 Age) + γ_12_ × (T0 Family atmosphere) + γ_13_ × (T0 Parenting style) + γ_14_ × (T0 Alienation toward parents) + γ_15_ × (T0 Life-events) + U_1_.

Hayes's PROCESS macro model was also applied to investigate the possible mediation of life-events in linking the baseline alienation toward parents and T4 depression (38). Bootstrap tests (2,000 repeated samples and 95% confidence interval) were used to test the significance of the mediating effect, with 95% CI did not contain 0 indicating a significant mediating effect. The data analyses were conducted using SPSS version 22 and HLM version 6.0. Also, *p* < 0.05 was considered as statistically significant.

### Control and Test of Common Method Deviation

The self-reporting information may cause deviations in the common method. Therefore, the procedural control was focused since the beginning, including applying mature scales with good reliability and validity, protecting the anonymity of participants and the reverse scoring of some items. Second, after the data collection, the common method deviation was tested with the one-factor test of Haman (39). The final results showed that during the five measures, the total numbers of factor with eigenvalues >1 were 15, 16, 15, 14, and 15, respectively, and the variances explained by the first factor were 20.10, 21.66, 23.54, 23.54, and 23.93%, and all were <40% for the critical standard (40). The results fully manifested that the deviations of the common method among five investigations were not significant.

## Results

### Descriptive Statistics

The demographic differences on the scores of alienation toward parents, life-events, and depression at baseline were summarized in Table 1. The scores of depression, alienation toward parents, and life-events did not differ significantly on gender, grade, or parents' marital status. However, they significantly differed in age, family atmosphere, and parenting style (*p* < 0.05). Specifically, the children aged 9 years experienced higher depression than the children with different age. The children from the family with frequent quarrels reported higher scores of alienation toward parents, life-events, and depression compared with those with occasional quarrel or harmonious family atmosphere (*p* < 0.001). Similarly, LBC with compulsive parenting style reported higher scores of alienation toward parents, life-events, and depression compared with those with other parenting styles (*p* < 0.001).

**Table 1:** Demographic characteristics of alienation toward parents, life-events, and depression at baseline [M(P_25_, P_45_)].

**Characteristic (877)** **[*n*(%)]**	**Alienation toward parents** **T0**	**Z**	**Life-events** **T0**	**Z**	**Depression** **T0**	**Z**
**Gender**						
Male [466 (53.14%)]	28 (23, 39)	−0.21	27 (15, 45)	−0.16	37 (33, 45)	−0.78
Female [411 (46.86%)]	29 (24, 37)		25 (14, 41)		38 (33, 43)	
**Grade**						
Four grade [344 (39.22%)]	28 (23, 38.75)	0.39	26 (15, 41)	0.62	38 (33.25, 44)	1.78
Five grade [280 (31.93%)]	29 (24, 37)		25 (13.25, 43)		37 (32, 43.75)	
Six grade [253 (28.85%)]	29 (23, 38)		27 (15.5, 43)		37 (33, 43)	
**Age**						
Nine years old [132 (15.05%)]	29.5 (24, 34)	6.66	28 (16, 41.50)	5.33	39 (34.25, 45.75)	9.26*
Ten years old [308 (35.12%)]	27 (22, 36)		24 (13, 41)		37 (32, 42)	
Eleven years old [262 (29.88%)]	29 (24, 40)		27.5 (16, 46)		38 (33.75, 44)	
Twelve years old [175 (19.95%)]	30 (23, 38)		27 (14, 41)		37 (33, 43)	
**Family atmosphere**						
Harmony [262 (29.88%)]	26 (22, 32)	58.25***	18.5 (9, 32)	83.99***	35 (31, 40)	83.77***
Occasional quarrel [567 (64.65%)]	29 (24, 39)		29 (17, 45)		38 (34, 44)	
Frequent quarrel [48 (5.47%)]	43 (31.5, 54)		51.5 (33.25, 65)		49 (43.25, 55)	
**Parenting style**						
Compulsive [200 (22.81%)]	32.5 (24, 45)	22.13***	31 (17, 49.75)	18.11***	41 (35, 48)	49.79***
Indulged [125 (14.25%)]	29 (23.5, 41.5)		30 (16.5, 47)		39 (34, 44.5)	
Spoiled [83 (9.46%)]	29 (22, 36)		25 (14, 40)		38 (35, 47)	
Democratic [469 (53.48%)]	27 (23, 36)		24 (13, 39)		36 (32, 41)	
**Parents' marital status**						
Un-divorced [663 (75.60%)]	28 (23, 37)	5.43	26 (14, 41)	2.00	38 (33, 44)	3.43
Divorced [114 (13.00%)]	30 (22.75, 39.25)		26 (14.75, 43)		38 (33, 43.25)	
Father remarried [27 (3.08%)]	34 (24, 49)		24 (15, 45)		37 (33, 43)	
Mother remarried [32 (3.65%)]	29 (25, 45.5)		25 (15.25, 56.75)		36 (32, 45.25)	
Both remarried [41 (4.67%)]	29 (22, 44.5)		29 (16, 48)		39 (36, 46.5)	

* *p < 0.05,*

*** *p < 0.001*.

As expected, the Spearman correlation analysis demonstrated that alienation toward parents, life-events, and depression were positively and moderately correlated with each other at the same or different time-points (*r* = 0.14 ~ 0.64, *p* < 0.001). See Table 2 for details.

**Table 2:** Correlations between depression, alienation toward parents, and life-events between five investigations.

	**M(P_**25**_, P_**45**_)**	**1**	**2**	**3**	**4**	**5**	**6**	**7**	**8**	**9**	**10**	**11**	**12**	**13**	**14**
1T0 Alienation	29 (23, 38)	1													
toward parents															
2T1 Alienation	26 (22, 35)	0.38***	1												
toward parents															
3T2 Alienation	26 (22, 36)	0.23***	0.31***	1											
toward parents															
4T3 Alienation	27 (22, 36)	0.40***	0.34***	0.26***	1										
toward parents															
5T4 Alienation	27 (22, 34)	0.34***	0.27***	0.22***	0.46***	1									
toward parents															
6T0 Life-events	26 (15, 42)	0.40***	0.28***	0.16***	0.31***	0.27***	1								
7T1 Life-events	22 (11, 39)	0.24***	0.42***	0.23***	0.25***	0.23***	0.54***	1							
8T2 Life-events	21 (10, 37)	0.17***	0.23***	0.44***	0.19***	0.18***	0.30***	0.47***	1						
9T3 Life-events	21 (10, 37)	0.30***	0.25***	0.21***	0.41***	0.28***	0.58***	0.48***	0.38***	1					
10T4 Life-events	21 (10, 38)	0.26***	0.21***	0.20***	0.31***	0.38***	0.53***	0.42***	0.34***	0.64***	1				
11T0 Depression	37 (33, 44)	0.43***	0.32***	0.17***	0.31***	0.25***	0.50***	0.32***	0.18***	0.36***	0.31***	1			
12T1 Depression	36 (32, 43)	0.25***	0.43***	0.25***	0.27***	0.20***	0.30***	0.50***	0.25***	0.32***	0.27***	0.51***	1		
13T2 Depression	36 (32, 43)	0.14***	0.27***	0.40***	0.18***	0.17***	0.19***	0.29***	0.49***	0.25***	0.23***	0.29***	0.42***	1	
14T3 Depression	36 (32, 44)	0.29***	0.25***	0.22***	0.43***	0.30***	0.42***	0.33***	0.19***	0.50***	0.42***	0.58***	0.46***	0.34***	
15T4 Depression	36 (31, 44)	0.26***	0.23***	0.21***	0.33***	0.44***	0.35^***^	0.34***	0.21***	0.39***	0.51***	0.48***	0.44***	0.30***	0.63***

*T0, T1, T2, T3, and T4 represent baseline, 1-month, 3-months, 6-months, and 12-months investigation, respectively;*

*** *p < 0.001*.

### Prediction of T0 Alienation Toward Parents and Life-Events on Developmental Trajectory of Depression

#### Null Model

The results of the Hierarchical Linear Model demonstrated that fixed effect and random effect of the model passed the significance test, and the intra- and inter-group variance were 41.57 and 29.52, respectively. Afterward, the inter-class correlation (ICC) was found to be 0.42. According to the former research (41), when ICC is <0.059, the hierarchy effects cannot be ignored between different levels, and a hierarchical linear analysis is of emphasis and necessity (37).

#### Unconditional Model

As listed in Table 3, the intercept of depression (B_0_ = 39.13, *t* = 138.76, *p* < 0.001), alienation toward parents (B_01_ = 31.73, *t* = 70.79, *p* < 0.001), life-events (B_02_ = 29.94, *t* = 40.96, *p* < 0.001), and the slope of depression (B_1_ = −0.23, *t* = −3.12, *p* < 0.01), alienation toward parents (B_11_ = −0.24, *t* = −2.04, *p* < 0.05), life-events (B_12_ = −0.86, *t* = −4.96, *p* < 0.001), were all significant. The results indicated that there was a linear downward trend for depression, alienation toward parents, and life-events over time. Moreover, the random effect indicated that there was a significant individual difference at the initial level of depression (χ^2^ = 1410.24, *p* < 0.001), alienation toward parents (χ^2^ = 1391.44, *p* < 0.001), and life-events (χ^2^ = 1556.61, *p* < 0.001), while the descendant rate also showed significant individual differences in depression (χ^2^ = 1060.48, *p* < 0.001), alienation toward parents (χ^2^ = 1053.58 *p* < 0.001), and life-events (χ^2^ = 955.26, *p* < 0.05). Thus, other potential influential variables could be introduced into the following full model.

**Table 3:** Estimated results of the unconditional model.

**Variables**	**Fixed effect**	**Coefficient**	**Standard error**	**T**
Depression	Intercept B_0_	39.13	0.28	138.76***
	Slope B_1_	−0.23	0.07	−3.12**
	Random effect	Variance	d	X^2^
	Intercept γ_00_	26.42	876	1410.24***
	Slope γ_10_	0.83	876	1060.48***
Alienation toward parents	Fixed effect	Coefficient	Standard error	t
	Intercept B_01_	31.73	0.45	70.79***
	Slope B_11_	−0.24	0.12	−2.04*
	Random effect	Variance	d	X^2^
	Interceptγ_01_	63.35	876	1391.44***
	Slope γ_11_	2.05	876	1053.58***
Life–events	Fixed effect	Coefficient	Standard error	t
	Intercept B_02_	29.94	0.73	40.96***
	Slope B_12_	−0.86	0.17	−4.96***
	Random effect	Variance	d	X^2^
	Intercept γ_02_	205.19	876	1556.61***
	Slope γ_12_	2.17	876	955.26^*^

* *p < 0.05,*

** *p < 0.01,*

*** *p < 0.001*.

#### Full Model

It can be seen in Table 4, the coefficients of γ_04_ = 1.85 (*p* < 0.001), and γ_05_ = 1.78 (*p* < 0.001) revealed that alienation toward parents and life-events at baseline positively associated with the intercept of depression. Furthermore, T0 life-events (γ_15_= 0.20, *p* < 0.05) positively predicted the descendant rate of depression, while the baseline alienation (γ_14_ = 0.09, *p* > 0.05) did not predict the decline of depression significantly. According to the random effect, the interaction between family atmosphere, parenting style, alienation toward parents, and life-events explained 9.64% [(0.83 – 0.75)/0.83] (42) of the total variation of the depression. The descendant trend of depression was explained 52.73% [(26.42 – 12.49)/26.42] by age and life-events.

**Table 4:** Estimated results of the full model.

**Fixed effect**	**Coefficient**	**Standard error**	**t**
Intertest Effect			
Intercept γ_00_	39.25	1.32	29.72***
T0 Age γ_01_	−0.43	0.25	−1.77
T0 Family atmosphere γ_02_	1.69	0.52	3.27**
T0 Parenting styleγ_03_	−0.67	0.21	−3.29**
T0 Alienation toward parents γ_04_	1.85	0.33	5.58***
T0 Life–events γ_05_	1.78	0.30	6.01***
Slope			
Time γ_10_	−0.38	0.37	−1.02
T0 Age γ_11_	0.15	0.07	2.13*
T0 Family atmosphere γ_12_	−0.28	0.15	−1.87
T0 Parenting styleγ_13_	0.09	0.06	1.52
T0 Alienation toward parents γ_14_	0.09	0.09	1.00
T0 Life–events γ_15_	0.20	0.09	2.29*
Random effect	Variance	d	R^2^
Intercept γ_00_	12.49	871	1122.16***
Slope γ_10_	0.75	871	1036.37***

* *p < 0.05,*

** *p < 0.01,*

*** *p < 0.001*.

### Mediation of Life-Events Between Alienation Toward Parents and Depression

**Figure 1:**
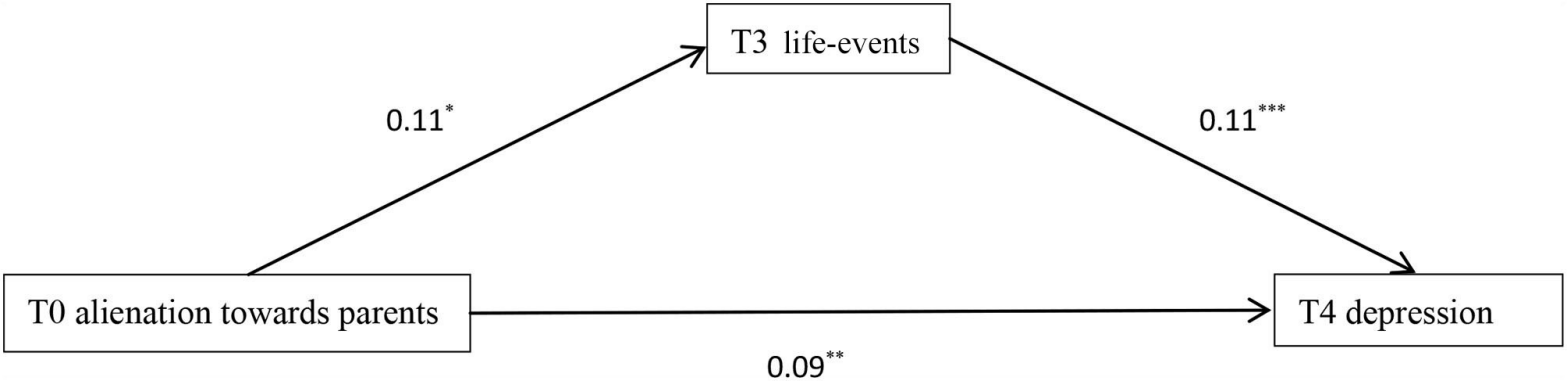
Mediation of T3 life-events in the prediction of T0 alienation toward parents on T4 depression. *p < 0.05, **p < 0.01, ***p < 0.001.

To further explore the causal relationship between alienation toward parents, life-events, and depression, Hayes's PROCESS was carried out. With T0 depression as controlled variable, T0 alienation toward parents as predictive variable, T1, T2, and T3 life-events as mediator separately, and T4 depression as dependent variable, the results showed that none of the mediation effects was significant. A further analysis was carried out to explore the potential mediation of life-events between alienation and later depression, with T1 and T2 alienation toward parents as predictive variable separately (T1 and T2 depression as controlled variable), T2 and T3 life-events as mediator separately, and T4 depression as dependent variable, the results showed that only T3 life-events totally mediated the prediction of T2 alienation toward parents on T4 depression (indirect effect = 0.03, 95% CI 0.01 – 0.05). With life-events at baseline as controlled variable, T0 alienation toward parents as predictive variable, T1, T2, and T3 life-events as mediator separately, and T4 depression as dependent variable, the results showed that T0 alienation toward parents had a direct effect on T4 depression (direct effect = 0.09, *p* < 0.01). Meanwhile, only T3 life-events partially medicated the prediction of T0 alienation toward parents on T4 depression (indirect effect = 0.07, 95% CI 0.01 – 0.02, *p* < 0.01) (Figure 1).

## Discussions

Depression is quite prevalent and becomes a significant issue among LBC (10). The present longitudinal study seems the first report of its kind, as far as we know, to explore the developmental trend of depression and the prediction of alienation toward parents on the developmental trajectory of depression in China's LBC and potential mediation of life-events. There were four essential findings in this study. First, alienation toward parents, life-events, and depression were positively and moderately related to each other at the same or different time-points. Second, there was a downward trend in depression, alienation toward parents, and life-events in LBC over time. Third, T0 alienation toward parents and T0 life-events positively predicted the developmental trajectory of depression over time. Fourth, T3 life-events mediated the prediction of baseline alienation toward parents on T4 depression in LBC.

The demographic information analysis showed that the age of LBC, family atmosphere, and parenting style influenced the scores of depression in LBC significantly. Specifically, the LBC aged 9 years tasted stronger depression than the children with other ages, which suggests that younger children may experience higher depression when their parents are leaving home. It was consistent with previous findings, which reported higher rates of depression in younger LBC (43). It could be explained by their irreplaceable role of parents in the development of younger children (14). Importantly, the children with family atmosphere of frequent quarrels and compulsive parenting style reported higher levels of alienation toward parents and depression, which proved consistent with the previous studies (17, 44, 45). Notably, inadequate parenting styles would lead to childhood abuse (46), which hurt parent–child relationship and increase alienation toward parents consequently (44). In daily life, almost all couples have sorts of conflicts, but only the children who really perceive family conflicts endanger their mental health (47). The children whose parents experienced divorce or involved in highly conflicting disputes (including ongoing conflicts) reported more alienation toward their parents (19). Furthermore, the current results revealed that frequent quarrel and compulsive parenting style increased the reports of life-events in LBC. The possible explanation may be that children with continual parental conflicts and compulsive parenting styles tend to adopt inadequate coping strategies when facing with life-events (48). Indeed, without a timely guidance and support from their parents, the LBC are susceptible to the effects of life-events. Together, the results suggest that elder age, harmonious family atmosphere, and democratic parenting style are vital protective factors for parent–child bond and children's mental health.

The Hierarchical Linear Model illustrated a downward trend of depression, alienation, and life-events in LBC, which was inconsistent with previous research which pointed out that the rate of youth depression was increasing year by year (49). The reason might be that, with the extension of left-behind duration, the proportion of LBC who understand the hard work and dedication of migrated parents increases (50), which stimulates their motivation to repay their parents through positive efforts in turn (51). In addition, as the children grow up, peers play an increasingly important role for them. To some extent, friendship can fill in the emotional vacancy gap for left-behind children to meet their needs for warmth and support (52), and results in a decrease in alienation toward parents, life-event, and depression. Moreover, our results indicated that life-events positively and independently predicted the descendant rate of depression, which indicated that the more the life-events, the faster the decline in depression declines (42). The reason maybe those adolescents who are experiencing more life-events may improve their coping abilities faster, thereby enhancing their psychological resilience effectively (53), and result in faster decline in depression (54). Besides, the baseline alienation did not predict the decline of depression significantly. There may be some factors protecting against the effect of alienation toward parents on the development of depression in high-risk LBC, such as friendship (52). Thus, in preventing and lowering the occurrence of depression in LBC, the knowledge helps to shape a better understanding about the depression in a more comprehensive and objective way (55).

The correlation analysis showed that alienation toward parents, life-events, and depression were positively correlated with each other. Moreover, the HLM showed that there was a positive prediction of alienation toward parents and life-events at baseline on the developmental trajectory of later depression in LBC, consistent with our hypothesis. It has been well reported that the children exposed to high alienation toward parents were related to higher likelihood of depressive symptoms and diminished health-related quality of life (19, 22). Our previous investigations also confirmed the connection between alienation, life-events, and depression in LBC (17, 18). The current longitudinal investigation further revealed the prediction of alienation and life-events on the developmental trajectory of later depression in LBC. This knowledge helps to better prevent the development of depression in LBC by paying attention to parent-child relationship (especially alienation toward parents) and children's life-events.

Importantly, this study further confirmed that T3 life-events partially mediated the prediction of baseline alienation toward parents on T4 depression in LBC, which manifested that life-events were important “bridge” linking alienation toward parents to depression. As a well-reported vulnerability for depression (56), this study confirmed the role of negative life-events in the mediation between alienation toward parents and depression. The indirect effect of life-events also implied that high levels of alienation toward parents during the childhood might make people difficulty to cope with negative life-events well, which aggravated the development of depression. Depression is a common negative emotion which occurs when people feel unable to cope with persistent external pressure (7). The children with high levels of alienation toward parents exhibited high levels of depression in the context of negative life-events (29, 57). Due to the limited emotional communication and support from parents, the LBC might taste more negative feelings when they encountered negative life-events, which put them at higher risks for depression (12). Based on the previous correlation findings from a cross-sectional study (6), the current results added to the growing body of knowledge about the predictive effect of alienation toward parents on depression in LBC when exposed to life-events. One point that deserves to point out is that, when controlled for baseline depression, the mediation of life-events between alienation toward parents and later depression was only significant for T3 life-events between the effect of T2 alienation on T4 depression. The findings indicate a close relationship or a sort of co-variation between alienation and depression in LBC, the mediation of life-events only exists when alienation has a weaker effect on depression and other variables, such as life-events, have stronger impact. The results suggest that to prevent the development of depression of LBC effectively, children with high alienation toward parents should be attended, especially when they experienced negative life-events, as well as the necessity to improve the capacity of coping with life-events well in LBC.

## Limitations

First, this study only used self-rating scales to evaluate the level of alienation toward parents, life-events, and depression, which may be subjected to self-report bias. Second, the participants came from three rural primary schools in Chongqing, China, so we should be very cautious to generate the results to other population. Third, some variables, such as friendship, may influence the development of depression in LBC, which was not included in this study, which might refrain the explaining power of our results. Fourth, when controlled for baseline depression, the mediation of life-events between alienation and depression only existed when alienation had a weaker effect on depression and other variables, such as life-events, had stronger impact, thus we should not assume that life-events mediate the prediction of alienation on depression all the time. However, the previous study only observed a connection between alienation toward parents and depression in LBC with cross-sectional design. With this longitudinal investigation, this study allowed us to explore the prediction of alienation on later depression, with a potential mediation of life-event.

In sum, this study is the first to suggest that alienation toward parents, although relatively overlooked in the existing literature, predicts the developmental trajectory of depression in LBC. Life-events partially mediate the prediction of alienation toward parents on later depression. These findings suggest that family and school may enhance the mental health of LBC by improving their parent–child relationship and offering more guidance to deal with life-events effectively. The government may reduce the numbers of LBC gradually by encouraging and guiding rural labors to make a successful living at hometown. In brief, the current findings provide reliable evidence for the prevention and intervention of depression in LBC.

## Data Availability Statement

The raw data supporting the conclusions of this article could be obtained from the corresponding author upon adequate requirement.

## Ethics Statement

The studies involving human participants were reviewed and approved by Human Research Ethics Committee, Army Medical University. Written informed consent to participate in this study was provided by the participants' legal guardian/next of kin.

## Author Contributions

XQ mainly wrote and revised the manuscript. XS revised the manuscript mainly for analysis. MZ, BC, FX, ZC, SS, CW, and XR collected the data together. QD designed the study. All authors contributed to the article and approved the submitted version.

## Funding

QD claimed that this study was supported by key project of natural science foundation of Chongqing (cstc2020jcyj-zdxmX0009), National Social Science Foundation of China (17XSH001), and the Key project and innovation project of People's Liberation Army of China (2021HL003).

## Conflict of Interest

The authors declare that the research was conducted in the absence of any commercial or financial relationships that could be construed as a potential conflict of interest.

## Publisher's Note

All claims expressed in this article are solely those of the authors and do not necessarily represent those of their affiliated organizations, or those of the publisher, the editors and the reviewers. Any product that may be evaluated in this article, or claim that may be made by its manufacturer, is not guaranteed or endorsed by the publisher.
